# Clinical evidence and implementation guidance for biosimilar switching in ankylosing spondylitis

**DOI:** 10.3389/fphar.2026.1803260

**Published:** 2026-04-15

**Authors:** Luoyang Cai, Xin Chen, Yangbin Fang, Li Li, Tao Liu, Zhenkun Gan, Zheng Zuo

**Affiliations:** Yunnan University of Chinese Medicine, Kunming, Yunnan, China

**Keywords:** ankylosing spondylitis, biosimilar pharmaceuticals, drug substitution, therapeutic equivalency, treatment outcome, tumor necrosis factor inhibitors

## Abstract

Biosimilar tumor necrosis factor inhibitors provide therapeutically equivalent alternatives to reference biologics for ankylosing spondylitis (AS) management at reduced costs. Clinical adoption depends on the confidence that switching stable patients from reference products to biosimilars maintains disease control. This review synthesizes the evidence on biosimilar switching in AS populations from regulatory approval programs, randomized controlled trials, and real-world observational studies. It is presented as a structured narrative review based on searches of PubMed, EMBASE, and the Cochrane Library, covering publications from January 2013 to March 2025. Approved biosimilars achieve molecular similarity and therapeutic equivalence to the reference products through analytical characterization, functional equivalence studies, and clinical comparability trials. Randomized switching trials demonstrate maintained efficacy and comparable safety profiles when patients with stable AS transition from the reference infliximab to biosimilar formulations. Immunogenicity profiles demonstrate equivalence between products. Multinational registry data corroborate these findings across diverse healthcare systems. Retention rates and disease activity outcomes are comparable between biosimilars and reference products when baseline patient characteristics are considered. Successful implementation requires patient engagement through transparent communication about biosimilar development and switching evidence. Individualized monitoring protocols should be tailored to baseline disease stability. Clinicians should recognize nocebo effects as a distinct contributor to switching outcomes and apply structured management strategies to address them. The accumulated evidence supports biosimilar switching as an appropriate clinical therapeutic strategy for AS patients with stable responses on reference biologics. This approach offers healthcare cost reductions without compromising treatment outcomes.

## Graphical Abstract

**Figure:**
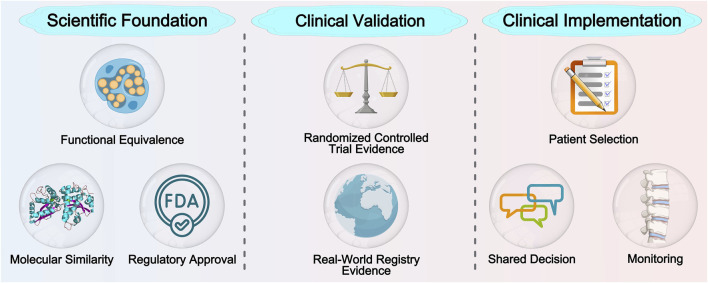

## 1 Introduction

Ankylosing spondylitis (AS) affects approximately 0.2%–0.5% of adult populations globally. Chronic inflammation of the axial skeleton causes progressive pain, stiffness, and functional limitation (Zhu et al., 2019). These manifestations substantially impair the quality of life and work productivity. Tumor necrosis factor (TNF) inhibitors revolutionized treatment by targeting inflammatory pathways directly. They achieve disease control beyond that possible with conventional therapies alone. Reference TNF inhibitors received regulatory approval for the treatment of AS between 2003 and 2016. These agents demonstrated substantial efficacy in both randomized trials and clinical practice. Approximately 60%–80% of AS patients achieve clinically meaningful responses (Mauro et al., 2021). Many sustain long-term disease control with continued therapy. Despite these therapeutic advances, access remains limited in many healthcare systems. The annual costs for reference TNF inhibitors range from 15,000 to 30,000 dollars per patient. These expenses consume substantial portions of rheumatology budgets (Abdelrahman and Mortada, 2020). Treatment costs restrict the prescription of biologics to patients with severe disease, who are unresponsive to conventional therapy. This creates treatment inequities where the medication costs rather than clinical appropriateness determine care access.

Biosimilar development emerged as a regulatory and scientific response to these challenges in care access. Regulatory pathways established by the European Medicines Agency (EMA) in 2005 and the United States Food and Drug Administration (FDA) in 2015 permit approval as they demonstrate biosimilarity (Lee et al., 2020). This requires analytical characterization, functional equivalence studies, and targeted clinical trials rather than complete development programs. Biosimilar TNF inhibitors entered European markets beginning in 2013 with infliximab biosimilar CT-P13. These products typically offer 15%–35% cost reductions compared to reference products (Kim et al., 2020d). Greater discounts become possible through competitive procurement in some healthcare systems. Economic modeling across multiple countries projects substantial cost savings from biosimilar inhibitor adoption. These savings could enable treatment access expansion for AS patients who are currently denied biologics due to budget constraints (Aapro et al., 2018). Realizing these economic benefits depends on clinical confidence that switching maintains disease control.

Several reviews have broadly examined biosimilar inhibitor adoption in rheumatic diseases. Earlier analyses covered multiple inflammatory conditions or focused on regulatory perspectives. There is existing disease-specific guidance for rheumatoid arthritis biosimilar switching where extensive trial data were accumulated earlier (Andrade et al., 2024; Cheng et al., 2025). However, AS-specific switching evidence has emerged predominantly since 2018. Major registry data were published between 2019 and 2024. Previous reviews either predated this evidence or addressed multiple conditions without AS-specific synthesis. There remain critical gaps regarding optimal patient selection beyond the simple disease stability criteria. The management of nocebo effects specific to AS populations requires clarification. Integration of real-world registry findings with trial evidence to inform implementation strategies remains incomplete. The current evidence base now permits such disease-specific synthesis.

We examined in this review whether AS patients who were stabilized on reference TNF inhibitors can safely transition to biosimilar formulations. This switching scenario differs from the biosimilarity determination established through regulatory approval. Regulatory approval confirms equivalence when biosimilar formulations are initiated in treatment-naive populations. Switching introduces additional considerations beyond the initial biosimilarity demonstration. Clinicians face a genuine dilemma when considering transitions. Stable AS patients have achieved disease control, often after trials of multiple therapies. Transitioning to a different product raises concerns about disrupting this stability. Maintaining efficacy in individual patients after the switch represents an important concern. Switching may also trigger new immunogenic responses that are not observed with continuous single-product exposure. The transition process itself could affect patient confidence and adherence. These concerns require examination through dedicated switching studies rather than extrapolation from biosimilarity data in naive cohorts.

This review addresses the clinical appropriateness of biosimilar switching in AS patients through evidence synthesis across multiple domains. We examine regulatory foundations that establish product similarity through analytical and functional characterization. We then evaluate whether this theoretical similarity translates to maintained clinical outcomes when switching stable patients. This review integrates evidence across study designs. Randomized trials provide controlled comparisons of switching versus continuation. Registry studies capture real-world effectiveness and safety across diverse populations and healthcare settings. We focus specifically on AS rather than spondyloarthritis broadly for several reasons. AS patients often require prolonged biologic therapy, making switching economics particularly relevant. Disease monitoring relies on specific validated instruments that differ from those of other conditions. The nocebo phenomenon may manifest differently given the chronic pain component that is intrinsic to AS. This disease-specific focus permits targeted guidance that is applicable to AS clinical practice. This review addresses practical implementation considerations that are often absent from trial reports. These include optimal patient-selection criteria, communication strategies to minimize nocebo effects, individualized monitoring protocols, and management of concerns arising post-switch. By synthesizing evidence across these domains, we provide clinicians with actionable guidance for implementing biosimilar switching decisions in their AS patients.

## 2 Literature search strategy

This article was prepared as a structured narrative review. A literature search was performed across PubMed, EMBASE, and the Cochrane Library, covering publications from January 2013 to March 2025. The starting date corresponds to the approval of the first infliximab biosimilar (CT-P13) by the EMA, which marked the beginning of the clinical use of biosimilars in AS patients. The search terms included “ankylosing spondylitis,” “biosimilar,” “biologic switching,” “TNF inhibitor,” “infliximab,” “etanercept,” “adalimumab,” “nocebo effect,” “drug retention,” and “real-world evidence,” which were combined using Boolean operators as appropriate.

Eligible sources included randomized controlled trials, prospective and retrospective cohort studies, multinational registry analyses, and conference abstracts reporting original data on biosimilar initiation or switching outcomes in AS populations. Clinical guidelines issued by the European League Against Rheumatism (EULAR) and the American College of Rheumatology (ACR), along with relevant regulatory documents, were additionally consulted to provide policy and clinical context. Sources were excluded if they did not report AS-specific outcomes, consisted solely of single case reports, or were unavailable in English. The reference lists of the included publications were manually screened to identify additional relevant sources that were not captured by the database search.

Two authors reviewed potentially eligible sources and reached a consensus through discussion. Given the heterogeneity of study designs and outcome reporting across the included publications, a formal meta-analysis was not performed. Evidence was instead synthesized narratively and organized by study design and clinical domain.

## 3 Scientific rationale for biosimilar switching

### 3.1 Molecular and functional equivalence supporting product interchangeability

Regulatory approval by the EMA and FDA confirms that biosimilar TNF inhibitors achieve high degrees of similarity to their reference products. This approval relies on evidence demonstrating molecular similarity, functional equivalence, and comparable clinical performance. The regulatory determination establishes that approved biosimilars and reference products are expected to produce the same clinical results in patients (Cordeiro et al., 2024). This foundation provides the scientific basis for considering product interchangeability in clinical practice. Understanding how regulatory processes establish equivalence clarifies why switching between molecularly similar products represents a scientifically sound strategy.

Multiple biosimilar TNF inhibitors have received regulatory approval for the treatment of AS with varying levels of switching evidence available (EMA, 2013; EMA, 2017a; EMA, 2017b; EMA, 2018; EMA, 2019; FDA, n.d.) (Table 1). Analytical characterization of these products utilizes orthogonal methods to compare biosimilar and reference products across multiple structural dimensions. Modern techniques including liquid chromatography–mass spectrometry, capillary electrophoresis, and circular dichroism spectroscopy resolve protein primary structures and higher-order conformations. Surface plasmon resonance evaluates the binding characteristics, while charge heterogeneity analysis examines post-translational modification patterns (Moots et al., 2017). These complementary approaches detect differences with high sensitivity. The approved biosimilar formulations demonstrate structural similarity within predefined acceptance ranges despite their production through independent manufacturing processes. For TNF inhibitors used in the treatment of AS, characterization of the infliximab biosimilar CT-P13, adalimumab biosimilar SB5, and etanercept biosimilar GP2015 confirmed that the physicochemical properties matched their respective reference products (Caporali et al., 2021). Detected microheterogeneity falls within ranges that do not affect the therapeutic function. Batch-to-batch variation exists even within reference product manufacturing, establishing that minor structural differences do not preclude therapeutic equivalence. The analytical evidence demonstrates that biosimilar formulations achieve the molecular similarity that is necessary to support interchangeable clinical use.

**TABLE 1:** Biosimilar products and evidence availability for AS.

Biosimilar product (trade names)	Reference product	Regulatory approval	Switching evidence level	Key studies	References
Infliximab CT-P13 (Remsima and Inflectra)	Infliximab (Remicade)	EMA (2013) and FDA (2016)	RCT and substantial registry data	PLANETAS extension, KOBIO registry, and ReFLECT	Borras Blasco et al. (2016), Park et al. (2017), Kim et al. (2020a), and Kim et al. (2020b)
Infliximab CT-P13 SC (Remsima SC)	Infliximab (Remicade)	EMA (2019)	Limited switching data, extrapolated from IV formulations	CT-P13 SC approval studies	Shirley (2021)
Infliximab GP1111 (Zessly, Ixifi)	Infliximab (Remicade)	EMA (2018), FDA (2018), and PMDA (2018)	Initiation studies, limited AS-specific switching data	GP1111 equivalence trials	Al-Salama (2018)
Etanercept GP2015 (Erelzi)	Etanercept (Enbrel)	EMA (2017) and FDA (2016)	Limited AS-specific switching data	GP2015 comparability studies	Deeks (2017)
Adalimumab SB5 (Imraldi)	Adalimumab (Humira)	EMA (2017) and FDA (2016)	Initiation studies, limited AS-specific switching data	SB5 equivalence trials and ASPIRE registry	Frampton (2018) and Kapoor et al. (2019)

Abbreviations: EMA, European Medicines Agency; FDA, United States Food and Drug Administration; IV, intravenous; PMDA, Pharmaceuticals and Medical Devices Agency; RCT, randomized controlled trial; SC, subcutaneous.

Functional assays determine whether the observed molecular similarity translates into equivalent biological activity relevant to AS treatment. TNF neutralization capacity represents the primary mechanism of action in AS pathophysiology. The key evaluations include receptor binding affinity, TNF activity neutralization, complement-dependent cytotoxicity, and antibody-dependent cell-mediated cytotoxicity (Emery et al., 2020). These *in vitro* assessments measure activities that directly correspond to the anti-inflammatory effects in AS. The etanercept biosimilar GP2015 demonstrated equivalent apoptosis inhibition in functional assays despite detectable molecular heterogeneity. This confirms that structural microheterogeneity within the regulatory acceptance ranges does not compromise the therapeutic function. Pharmacodynamic studies in humans provide additional validation. Both the etanercept biosimilar GP2015 and the adalimumab biosimilar SB5 exhibited pharmacodynamic properties that matched those of their reference products in human subjects (Deeks, 2017; Frampton, 2018). Clinical pharmacokinetic studies establish that biosimilars achieve equivalent systemic exposure. Population analyses demonstrate comparable concentration–time profiles, supporting equivalent drug availability at sites of inflammation. The convergence of analytical characterization, functional equivalence, and pharmacokinetic comparability establishes that TNF inhibition caused by biosimilars is equivalent to that produced by reference products.

The demonstrated molecular and functional equivalence between the biosimilar formulations and reference products supports a logical inference regarding switching scenarios. An AS patient who has achieved stable disease control on a reference TNF inhibitor responds to the specific molecular and functional properties of that product (Garcia-Montoya and Emery, 2021). When a biosimilar demonstrates equivalent molecular structure, equivalent functional activity, and comparable pharmacokinetics, it delivers the same therapeutic mechanism. The patient’s disease pathophysiology and treatment target remain unchanged during transition. Only the manufacturing source of the molecularly equivalent protein changes. Therefore, the maintained therapeutic response represents the expected outcome when switching from the reference product to the biosimilar. This reasoning applies specifically to patients with established stability, where the treatment has already demonstrated effectiveness. The biological rationale predicts that equivalent products should produce equivalent results in the same patient. This theoretical foundation requires validation through actual clinical switching studies.

One aspect requiring particular attention in switching scenarios involves immunogenic responses. While molecular and functional equivalence supports maintained efficacy, the immune system recognition of therapeutic proteins operates through complex mechanisms (Wroński et al., 2019). Product-related factors including aggregation propensity and formulation components can influence immunogenicity. Patient-related factors including the genetic background and immune system status also affect antibody formation. Whether switching between highly similar products alters the immunogenic risk compared to continued single-product use represents a testable hypothesis. Examining the theoretical basis for immunogenic equivalence clarifies the expectations for switching outcomes.

### 3.2 Immunogenic equivalence as a prerequisite for safe switching

All biologic therapies used in AS carry an inherent immunogenic potential due to their protein nature. Anti-drug antibody formation represents an expected biological response to foreign protein introduction (Vogan, 2023). Neutralizing antibodies that block the therapeutic mechanism represent the most concerning immunogenic consequence. They directly compromise treatment efficacy by preventing engagement with TNF targets. This can cause treatment failure in previously responding AS patients. Non-neutralizing antibodies affect clinical outcomes through pharmacokinetic mechanisms. They form immune complexes that accelerate drug clearance through the reticuloendothelial system. This reduces drug exposure below therapeutic thresholds even when the antibody does not block TNF binding (Kwon et al., 2025). However, detectable antibodies do not uniformly predict clinical consequences. Low-titer antibodies may have negligible functional effects. Individual patients vary substantially in the threshold antibody levels required to compromise the therapeutic benefit. The relationship between immunogenicity measurements and clinical outcomes in AS involves complex interactions between antibody characteristics and patient-specific factors.

Product-related factors that influence immunogenicity are highly similar between the approved biosimilars and their reference products. The protein sequence determines the potential for immune recognition through T-cell epitope presentation (Huang et al., 2024). Biosimilars of monoclonal antibodies such as infliximab and adalimumab share identical primary sequences with the reference products. This eliminates differences in the fundamental immunogenic potential from the amino acid composition. Etanercept biosimilars replicate the fusion protein structure of the reference etanercept. Post-translational modifications including glycosylation patterns affect immunogenic risk through altered protein conformation or aggregation propensity. Regulatory approval requires demonstrating that glycan profiles fall within acceptable similarity ranges. Formulation components such as excipients, buffers, and stabilizers undergo evaluation for potential immunogenic contributions. The approved biosimilars use formulations that maintain stability without introducing novel immunogenic stimuli (Liu et al., 2021). Aggregation represents an important factor because protein aggregates enhance immune recognition. The manufacturing process controls ensure that the aggregation levels remain within specifications that are comparable to those of the reference products. The similarity in product-related immunogenic determinants predicts equivalent immunogenic behavior between biosimilars and the reference products under identical use conditions.

Patient-related factors that modulate immunogenic responses remain constant during switching events in individual patients. The genetic background, particularly human leukocyte antigen haplotypes, influences T-cell-mediated antibody responses to therapeutic proteins. These genetic determinants do not change when a patient switches products. The immune system activation state affects responsiveness to foreign proteins (Britanova et al., 2023). An AS patient’s baseline inflammatory status and immune competence remain the same immediately before and after switching. Concomitant immunosuppressive medications including methotrexate or corticosteroids reduce anti-drug antibody formation risk. Continuation of stable concomitant therapy during switching maintains consistent immunomodulation. Prior exposure to the same protein class may induce immune tolerance in some patients or prime for enhanced responses in others. A patient switching from the reference infliximab to an infliximab biosimilar has already been exposed to the infliximab protein. This prior exposure history persists regardless of the manufacturing source (Tavasolian et al., 2023). Modifiable factors such as smoking status affect immunogenicity, but these behaviors typically remain stable over the short term surrounding a switch. The constancy of patient-level immunogenic determinants during product transition supports the expectation of stable immunogenic risk.

The timing of switching introduces an additional consideration regarding the established immune responses. Patients who remain on the reference products long enough to be considered for switching have typically received treatment for months or years. If these patients have not developed anti-drug antibodies during this exposure period, they have likely established immune tolerance to the therapeutic protein. Switching to a biosimilar with a highly similar molecular structure should not disrupt this established tolerance (Hunter et al., 2019). The immune system has already determined that the protein is acceptable. Introducing the same protein from a different manufacturing source does not present novel epitopes requiring new immune evaluation. Conversely, patients who have developed anti-drug antibodies for the reference products represent a different scenario. These antibodies typically recognize epitopes present in the protein sequence or conformational structure. Because biosimilars share these structural features, the existing antibodies would recognize the biosimilar products similarly to the reference products (Bhushan et al., 2021). Switching neither eliminates the existing antibodies nor introduces new immunogenic challenges. This analysis suggests that switching timing after immune tolerance establishment should not increase immunogenic risk compared to continued reference product exposure.

The theoretical analysis based on product similarity and patient factor constancy predicts that switching should not increase the immunogenic risk. Product-related determinants of immunogenicity achieve equivalence through regulatory requirements. Patient-related determinants remain unchanged during the switching process itself. The combination of equivalent product factors and stable patient factors supports the expectation of equivalent immunogenic outcomes. This prediction requires empirical validation through clinical studies that directly measure immunogenic responses in populations undergoing switching. Clinical evidence demonstrating comparable immunogenicity between switched and non-switched patients would validate the scientific rationale. Divergent immunogenic outcomes would indicate factors that were not captured by the theoretical analysis. Integration of theoretical predictions with empirical observations provides the complete foundation for assessing switching safety.

## 4 Empirical validation of switching outcomes in AS

### 4.1 Randomized controlled trial evidence

The theoretical predictions established above require empirical validation through clinical switching studies. Here, we examine whether randomized trials support three key expectations. First, switching should maintain disease control achieved by reference products. Second, switching should not compromise the safety profiles. Third, immunogenic responses should remain equivalent between switched and maintained patients. These predictions guide the evaluation of the available trial evidence in AS populations, as summarized in Table 2. The PLANETAS extension study provides the most methodologically rigorous switching evidence available for AS.

**TABLE 2:** Randomized controlled switching trials in AS.

Study (design, duration)	Treatment groups	Efficacy outcomes	Immunogenicity	Safety and discontinuation
Park et al., 2017 (randomized OLE; 102 weeks total)	CT-P13 maintenance (n = 88) vs. switched to CT-P13 from reference (n = 86)	ASAS-20 response 80.7% vs. 76.9%; ASAS-40 and partial remission similar; BASDAI well-controlled in both groups	ADA positivity was 23.3% vs. 27.4% at week 102	AE-related discontinuation was 3.3% vs. 4.8%; no unexpected events; safety profile comparable
Kaltsonoudis et al., 2019 (randomized parallel; 18 months)	Switched to biosimilar (n = 45) vs. continued reference (n = 43)	All patients in both groups maintained clinical remission throughout 18 months	Not separately reported for the AS subgroup	No significant AE between groups; five discontinued in the biosimilar group and three in the reference group
Benucci et al., 2017 (prospective observational; 6 months)	Switched from reference to biosimilar (n = 41 SpA patients including AS)	No changes in BASDAI (2.73 ± 1.5 vs. 2.6 ± 1.3), BASFI, and ASDAS-CRP after switching	No changes in circulating infliximab or anti-infliximab antibody levels	Very low number of AEs; one patient (3%) discontinued due to severe palmoplantar psoriasis
Batticciotto et al., 2016 (observational; 6 months)	Switched from reference to biosimilar (n = 31 SpA patients including AS)	No statistical differences in BASDAI, BASFI, ASDAS-CRP, or inflammatory markers	No reported changes in antibody levels	Low AE rate; no significant safety concerns

Abbreviations: ADA, anti-drug antibodies; AE, adverse events; ASAS, Assessment of SpondyloArthritis international Society; ASDAS, Ankylosing Spondylitis Disease Activity Score; BASDAI, Bath Ankylosing Spondylitis Disease Activity Index; BASFI, Bath Ankylosing Spondylitis Functional Index; CRP, C-reactive protein; OLE, open-label extension; SpA, spondyloarthritis.

Studies have shown that the initial 54-week trial established equivalence between the infliximab biosimilar CT-P13 and the reference infliximab in 250 treatment-naive AS patients (Park et al., 2016). At week 54, investigators offered enrollment in an open-label extension where patients originally assigned to the reference infliximab switched to CT-P13. Those originally assigned to CT-P13 continued biosimilar therapy. Among 174 patients entering the extension, 88 maintained CT-P13 throughout 102 weeks and 86 switched from the reference infliximab at week 54. The randomized initial assignment, prospective design, and standardized outcome assessments strengthen the internal validity of the switching comparisons. The Bath Ankylosing Spondylitis Disease Activity Index (BASDAI) scores at week 102 showed comparable disease control between groups. The maintenance group achieved an 80.7% Assessment of SpondyloArthritis International Society (ASAS)-20 response, while the switching group achieved 76.9% response. The ASAS-40 responses and ASAS partial remission rates were similar between groups (Park et al., 2017). These findings demonstrate that transitioning from the reference infliximab to the biosimilar preserved therapeutic control in patients who had achieved stable responses. Pharmacokinetic analyses measured trough infliximab concentrations serially through week 102. The levels showed comparability between groups without any evidence of reduced exposure following transition. Adverse event profiles aligned with the expectations for TNF inhibitor therapy. Adverse events leading to discontinuation occurred in 3.3% of the maintenance group and 4.8% of the switch group. Anti-drug antibody positivity rates at week 102 were 23.3% in the maintenance group and 27.4% in the switch group, confirming that switching did not increase immunogenic risk. The extension phase employed an open-label design, thus introducing potential bias through patient and physician awareness of treatment assignment. This limitation is particularly relevant for BASDAI, which relies entirely on the patient self-report and is, therefore, susceptible to expectation-driven response shifts in unblinded conditions. The authors themselves acknowledged that the extension was neither designed nor powered to formally evaluate non-inferiority or equivalence between the switch and maintenance groups. Therefore, the BASDAI comparability data should be interpreted with appropriate caution. Notably, pharmacodynamic analyses demonstrated comparable changes in the C-reactive protein (CRP) and erythrocyte sedimentation rate between groups at both week 54 and week 102 regardless of the anti-drug antibody status. This concordance between subjective disease activity scores and objective inflammatory markers provides partial reassurance that the open-label design did not substantially distort the overall efficacy findings.

Additional controlled switching studies provide supporting evidence across different settings and patient populations. An 18-month randomized study in Greece enrolled 88 AS patients in clinical remission on the reference infliximab, with 45 randomized to switch to a biosimilar and 43 to continue with the reference product. All patients who completed the study maintained clinical remission throughout 18 months of follow-up, with BASDAI and ASDAS-CRP showing no significant differences between groups. ESR and CRP values remained stable and comparable between groups at both the time of switching and study completion, providing objective inflammatory marker corroboration of the clinical remission data. This consistency between patient-reported and laboratory-based outcomes partially mitigates the potential bias introduced by the open-label design. Several methodological limitations warrant consideration when interpreting these findings. The authors acknowledged that the small sample size represents the primary limitation of the study, and no formal power calculation was reported, leaving the statistical capacity to detect small but clinically meaningful differences uncertain. The final efficacy analysis was based on completers rather than an intention-to-treat population. Five patients in the biosimilar group and three in the reference group discontinued during follow-up, with four biosimilar discontinuations attributable to nocebo effects manifesting as subjective complaints without corresponding objective disease activity changes. Excluding these patients before outcome analysis may have contributed to the uniformly favorable remission results observed at study completion. Within these constraints, the consistency between PLANETAS findings and those of this study across different baseline disease activity levels provides additional support for switching appropriateness in optimally controlled AS patients (Kaltsonoudis et al., 2019). Smaller observational studies with prospective designs contribute consistency signals despite methodological limitations. A 6-month Italian multicenter study followed 41 spondyloarthritis patients, including those with AS who switched from the reference to the infliximab biosimilar. Investigators found no significant changes in BASDAI, the Bath Ankylosing Spondylitis Functional Index (BASFI), or ASDAS after transition (Benucci et al., 2017). Another Italian study across three centers examined 31 patients, with similar findings of stable disease activity (Batticciotto et al., 2016). These studies enrolled patients with inactive or moderate disease activity at baseline. The preservation of disease control after switching in these real-world settings corroborates the trial findings despite smaller sample sizes and shorter follow-up periods.

Evidence for biosimilar switching with other TNF inhibitors is more limited than that for infliximab. A cohort study evaluating etanercept biosimilar switching included 13 AS patients among 87 total inflammatory arthritis patients (Ditto et al., 2020). The study found no efficacy differences between the reference and biosimilar products measured by objective disease activity parameters. However, some patients reported subjective symptoms after switching without any corresponding objective disease activity changes. This pattern indicates that nocebo effects may influence the perceived outcomes despite maintained biological disease control. The small number of AS patients in this study limits definitive conclusions about etanercept biosimilar switching. Published switching evidence for adalimumab biosimilars in AS populations is similarly sparse. The concentration of high-quality switching evidence for infliximab reflects both the earlier market entry of infliximab biosimilars and the prioritization of infliximab for research on switching. Direct switching evidence in AS varies substantially across TNF inhibitor agents. For the infliximab biosimilar CT-P13, the evidence base encompasses randomized controlled trial data and extensive multinational registry analyses, constituting the most robust foundation that is currently available. For other infliximab biosimilars, evidence derives from regulatory extrapolation based on CT-P13 comparability data rather than AS-specific switching studies. For etanercept and adalimumab biosimilars, published data on switching in AS populations remain sparse. Available reports involve either mixed inflammatory arthritis cohorts with small AS subgroups or observation periods that are insufficient to support definitive conclusions. Shared TNF neutralization mechanisms provide a theoretical basis for anticipating comparable switching behavior across agents. However, mechanistic similarity does not substitute for direct clinical evidence of equivalent switching outcomes. Clinical confidence should, therefore, be calibrated to the strength of the available evidence for each individual agent.

### 4.2 Real-world registry and observational evidence

Real-world studies provide important complementary evidence by evaluating biosimilar switching under conditions reflecting routine clinical practice. These studies include patient populations that trials typically exclude, such as elderly individuals, those with multiple comorbidities, patients with prior exposure to multiple TNF inhibitors, and individuals receiving concomitant therapies. Observation periods in registry studies often extend beyond typical trial durations, allowing the detection of delayed effects that are not apparent in shorter controlled trials. Several countries have established prospective biologics registries tracking patients receiving TNF inhibitors for AS (Rahman et al., 2016; Guo et al., 2021; Ko and Moon, 2025). These registries support biosimilar evaluation at the population scale, with sample sizes exceeding those feasible in randomized trials.

The Korean College of Rheumatology Biologics registry generated extensive evidence through complementary analyses addressing different aspects of use of biosimilars in AS treatment. An initial analysis followed 244 patients receiving the infliximab biosimilar CT-P13 for up to 4 years, including both biosimilar-naive initiators and patients who switched from the reference infliximab. The 4-year retention rate reached 66%, with BASDAI remaining well-controlled throughout follow-up (Kim et al., 2020a). Treatment changes occurred in 15.6% of patients, while discontinuations occurred in 13.1%. Lack of efficacy represented the most common reason for treatment changes, whereas adverse events were the most common cause of discontinuation. These retention rates reflect real-world persistence where patient adherence, medication access, and clinical decision-making all influence the outcomes beyond controlled trial conditions. A subsequent propensity score-matched analysis compared outcomes between 124 patients receiving the biosimilar and 124 matched controls receiving the reference infliximab. This analytical approach balanced baseline characteristics between the comparison groups to address potential selection bias where clinicians might preferentially switch patients with more stable disease, seeking to reduce confounding from preferential switching of more clinically stable patients (Kim et al., 2020b). The matching model incorporated the age, sex, and baseline BASDAI score. Disease duration, inflammatory markers, treatment line, and concomitant medication use were not included in the matching procedure. The matched analysis found 3-year retention rates of 64.2% for the biosimilar and 55.6% for the reference product without statistically significant differences. Despite these overall findings, residual baseline imbalances persisted within treatment-line subgroups after matching. In the first-line subgroup, the mean disease duration remained significantly longer in the CT-P13 group than in the reference infliximab group. In the subsequent-line subgroup, the mean ASDAS-CRP and CRP levels were higher in the CT-P13 group at baseline. The median CRP values also differed significantly between groups at 2, 3, and 4 years of follow-up. The authors noted that CRP measurement methods were not standardized across the 44 participating centers, which may have contributed to this divergence. Efficacy data were not collected for all patients, and some treatment discontinuations were recorded without a documented reason. These methodological features indicate that the matched groups may not have been fully equivalent on all clinically relevant prognostic variables. The retention findings should, therefore, be interpreted as supporting, rather than definitively establishing, equivalent long-term outcomes between the two treatments. Focused analysis of dose and interval modifications examined 337 AS patients who were followed-up for 5 years. Approximately half of the patients had treatment pattern changes involving dose or infusion interval adjustments. Patients with treatment pattern changes showed greater improvements in BASDAI scores compared to those without changes, indicating that individualized dosing optimization benefited clinical outcomes. Drug survival did not differ significantly between the patients with or without treatment pattern changes (Lee et al., 2021). These converging findings from a single robust registry across multiple analytical approaches provide real-world evidence supporting use of biosimilars in AS.

Registry data from multiple healthcare systems spanning Europe, Asia, and other regions provide multinational validation of biosimilar switching outcomes across different treatment contexts (Table 3). The French ReFLECT study followed 411 patients with rheumatic diseases, including AS, who received the biosimilar CT-P13. Among these, 179 had switched from the reference infliximab, while 228 were biosimilar-naive. The 24-month retention rate reached 74.9%, with the inflammatory markers and disease activity remaining stable throughout the follow-up period (Borras Blasco et al., 2016). This observation period extended substantially beyond most randomized trial follow-ups, addressing questions about longer-term switching outcomes. Both the switched and naive groups achieved comparable disease control by 24 months, with the median BASDAI scores of 2 in switched patients and 3 in naive patients. The Indian ASPIRE registry reported outcomes in 308 AS patients initiating the biosimilar adalimumab. The mean BASDAI decreased from 6.2 at baseline to 2.1 over 24 weeks. Low disease activity, which is defined as a BASDAI score below 4, was achieved by 94% of the patients (Kapoor et al., 2019). The Turkish TURKBIO registry examined 179 patients receiving CT-P13 as the first-line or second-line therapy, with retention rates of 58.6% for first-line and 48.2% for second-line use over 3 years (Uslu et al., 2024). The Nordic collaborative analysis pooled data across Denmark, Finland, Iceland, Norway, and Sweden, including 2,334 biologic-naive spondyloarthritis patients initiating either infliximab or etanercept. Treatment retention showed no significant differences between the biosimilars and reference products for either agent (Lindström et al., 2019). The consistency of favorable trends across these geographically and organizationally diverse registries strengthens confidence in generalizability despite methodological limitations that are inherent to observational research.

**TABLE 3:** Real-world registry studies of biosimilar use in AS.

Study (country, design)	Patients and follow-up	Retention rate	Clinical outcomes	Safety profile
KOBIO Registry (Korea; prospective cohort)	244 patients (203 naïve and 41 switched); 48 months	66% at 48 months	BASDAI decreased and remained stable; disease activity well-controlled	AE rate was 48.4% (94.6% mild to moderate); infusion reactions 4.1%, uveitis 3.7%
KOBIO PSM Analysis (Korea; propensity-matched)	248 patients (124 CT-P13, 124 reference); 36 months	CT-P13 64.2% vs. reference 55.6% (p = NS)	Efficacy parameters similar between groups	Comparable safety profiles between groups
ReFLECT Study (France; prospective observational)	411 patients (228 naïve and 179 switched); 24 months	74.9% at 24 months	CRP and disease activity stable in switched patients; improvement in naive patients	No new safety concerns identified
Nordic Collaborative (Denmark, Finland, Iceland, Norway, and Sweden; observational)	1,319 infliximab patients (biologic-naive); 24 months for infliximab	Biosimilar 46% vs. originator 44% at 24 months (p = NS)	Disease activity outcomes not separately reported in this analysis	Safety profiles similar between products
ASPIRE Registry (India; observational)	308 patients (biosimilar initiation); 24 weeks	Not reported	BASDAI decreased from 6.2 to 2.1; 94% achieved low disease activity (BASDAI<4)	Well tolerated; no new unexpected adverse reactions
TURKBIO Registry (Turkey; observational cohort)	179 patients (123 first-line, 56 ≥second-line); 36 months	First-line 58.6%; second-line or later 48.2%	ASAS-20/40 responses higher in first-line at 3 and 6 months; comparable at 12 months	Acceptable long-term safety profile
Eastern European Multicenter (Bulgaria, Czech Republic, and Romania; observational)	70 patients (biosimilar initiation); 24 weeks	Not reported	BASDAI and CRP significantly improved from baseline	Well-tolerated with a good safety profile
Italian Multicenter (Italy; prospective switching study)	41 patients (switched from originator); 6 months	96.8% (one patient discontinued)	No changes in BASDAI, BASFI, ASDAS-CRP, or inflammatory markers	Low AE rate; 3% (one patient) discontinued

Abbreviations: AE, adverse event; ASDAS, Ankylosing Spondylitis Disease Activity Score; BASDAI, Bath Ankylosing Spondylitis Disease Activity Index; BASFI, Bath Ankylosing Spondylitis Functional Index; CRP, C-reactive protein; NS, not significant; PSM, propensity score-matched.

Important insights regarding factors affecting switching outcomes emerged from analyses of implementation strategies. Dutch mandatory switching programs demonstrated that approximately one-quarter of patients discontinued biosimilar therapy within 6 months when switching occurred as a payer-mandated policy without individualized clinical assessment. Detailed analysis revealed that discontinuations were often associated with subjective symptom increases without corresponding changes in inflammatory markers or physician assessments (Tweehuysen et al., 2018). This dissociation pattern indicates that patient expectations and the manner in which switching is introduced exert a measurable influence on the retention outcomes independent of pharmacological factors (Boone et al., 2018). Studies conducted in settings emphasizing shared decision-making and patient education reported lower discontinuation rates compared to non-medical switches implemented through administrative mandate. This contrast demonstrates that successful biosimilar switching depends not only on biological product equivalence but also on patient engagement and clinical involvement in decisions on transition. The neurobiological mechanisms underlying these patterns and evidence-based strategies for managing this phenomenon are discussed below in the context of clinical implementation.

### 4.3 Safety profile and adverse events across studies

Safety evaluation addresses a fundamental question regarding biosimilar switching. A key question is whether transitioning from reference products to biosimilars introduces safety risks beyond those associated with continued use of the reference product. If biosimilars possess the same molecular structure and biological activity as the reference products, the safety profile should remain equivalent. This expectation applies regardless of whether the patients initiate biosimilar therapy or switch from reference products.

Pooled long-term safety analysis of the infliximab biosimilar CT-P13 included data from observational studies across multiple countries and indications. Among 1,389 patients with various inflammatory conditions including AS who received CT-P13, safety profiles were consistent with the TNF inhibitor class effects. The incidence and types of adverse events aligned with the established safety experience accumulated over 2 decades of reference infliximab use(Cheon et al., 2021). Treatment-emergent adverse events occurred at rates that are comparable to historical data, with most events classified as mild to moderate in severity. This convergence indicates that biosimilar exposure does not generate novel safety signals that are distinct from the reference product experience. A 5-year retrospective analysis conducted specifically in AS patients treated with CT-P13 found adverse events in 29.4% of patients. The most common treatment-related events included infusion reactions, uveitis, and skin rash, which are all recognized complications of TNF inhibition (Kim et al., 2020c). Serious adverse events deemed potentially drug-related occurred in 1.8% of patients, with tuberculosis representing the most concerning serious infection. These rates fall within the expected ranges based on the TNF inhibitor mechanism and regional epidemiology rather than indicating biosimilar-specific risks.

Real-world evidence from diverse geographic regions corroborated trial safety findings at a larger scale and longer duration. Eastern European multicenter trial experience following 70 AS patients initiating biosimilar infliximab for 24 weeks reported good tolerability without unexpected adverse events (Codreanu et al., 2018). The consistency of the safety observations across controlled trials, registry studies, and real-world cohorts spanning multiple countries demonstrates that biosimilar safety characteristics generalize across different patient populations and healthcare settings. The rates of serious infections requiring hospitalization were low without differences between the biosimilars and reference products when comparing matched populations. Malignancy incidence in studies with follow-up extending to 4 or 5 years remained within the expected ranges based on the age-matched population data and historical TNF inhibitor cohort experience. No specific malignancy types emerged preferentially in biosimilar-exposed populations, indicating that the theoretical immunosuppressive effects of TNF inhibition operate similarly regardless of the manufacturing source of the product.

The accumulated safety evidence supports the theoretical prediction that molecular and functional equivalence between the biosimilars and reference products translates into equivalent safety profiles. Biosimilar switching does not increase the overall adverse event burden beyond that associated with continued reference product use. No new safety signals specific to biosimilars or to the switching process have emerged despite extensive post-marketing surveillance involving thousands of patients across multiple countries. Long-term safety data extending to 5 years demonstrate sustained safety without late-emerging concerns that might not appear in shorter trials. This consistency across study designs, observation periods, and geographic regions provides confidence in switching decisions. Clinicians can proceed based on the same safety considerations that guide TNF inhibitor use generally.

## 5 Clinical implementation of biosimilar switching

### 5.1 Clinical decision-making for switching

Translating evidence into practice requires structured approaches for patient selection and clinical decision-making. The accumulated evidence supports switching in appropriately selected patients, but individual assessment remains essential to optimize the outcomes. Suitable candidates demonstrate sustained disease control defined by measurable criteria rather than subjective impressions. BASDAI should remain consistently below 4 over at least 3 consecutive months without recent fluctuations. The 2022 ASAS-EULAR recommendations for the management of axSpA define high disease activity as an ASDAS of 2.1 or above or, alternatively, a BASDAI of 4 or above when ASDAS is unavailable, providing the validated clinical thresholds against which switching candidacy can be assessed (Ramiro et al., 2023). Current biologic dosing and infusion intervals should have remained stable for at least 6 months without adjustments for disease activity. Inflammatory markers including CRP should show stable low levels corresponding to disease quiescence. Physical examination should document stable spinal mobility measurements and the absence of new peripheral joint involvement when relevant (Li et al., 2024). This stability window ensures that the observed post-switch outcomes reflect actual treatment effects rather than natural disease variability. Patients experiencing recent flares, undergoing dose modifications, or showing activity fluctuations should delay transition until stability is reestablished. The evidence base derives predominantly from studies enrolling patients meeting these stability criteria, making disease control the primary selection criterion supported by data.

Several clinical situations require individualized assessment beyond simple disease stability verification. Pregnancy planning or active pregnancy warrants careful timing consideration given the importance of maintaining stable control for maternal and fetal outcomes. Pregnancy-specific data for biosimilars are limited despite the absence of specific safety signals in available case reports. Patients stable on reference products who are planning pregnancy may benefit from avoiding switching during pregnancy or immediately before conception to minimize uncertainty during this sensitive period (Mokbel et al., 2021). Women who have already established biosimilar therapy before pregnancy can reasonably continue based on the general equivalence evidence. Patients with a history of severe uveitis or inflammatory bowel disease requiring combination therapy may benefit from particularly careful consideration of switching timing (Lai et al., 2021; Lee et al., 2025). Elderly patients and those with significant comorbidities including cardiovascular disease, chronic kidney disease, or diabetes mellitus can undergo switching during stable axial disease (Baniaamam et al., 2021; Liu et al., 2024). The presence of comorbidities does not contraindicate switching but requires thorough assessment, ensuring that the monitoring plans account for multiple concurrent conditions.

Shared decision-making forms the foundation of successful implementation by engaging patients as active participants in treatment transitions. Communication should explain that regulatory approval has confirmed molecular similarity and functional equivalence between the products through rigorous evaluation. Both clinical trials and real-world studies demonstrate maintained disease control after switching in patients with stable responses. Framing switching as a clinical decision supported by scientific evidence, rather than a purely administrative mandate, establishes appropriate expectations and reduces patient concerns about receiving inferior treatment. This approach is consistent with the position adopted by major rheumatology guidelines, which recognize biosimilars as equivalent to originator biologics for treatment purposes (Kay et al., 2018; Fraenkel et al., 2021). Specific discussion points should address three key areas that patients typically raise (Kwon et al., 2018). First, biosimilars undergo rigorous characterization demonstrating high similarity to the reference products despite independent manufacturing. Second, regulatory agencies require evidence of equivalent clinical performance within predefined margins excluding meaningful differences. Third, extensive experience across thousands of patients shows comparable effectiveness and safety between products. Patients should understand that their current disease control is expected to continue, with monitoring protocols in place to confirm this outcome. Transparent acknowledgment that some patients report symptom changes after switching and that structured approaches exist to evaluate and address these responses helps frame realistic expectations and supports patient confidence in the transition.

### 5.2 Implementation and follow-up

Monitoring intensity and duration should be tailored to individual patient characteristics rather than applying uniform protocols. Patients with longstanding stable disease may require only routine monitoring comparable to their pre-switch schedule. Those with recent disease activity fluctuations or complex comorbidities may benefit from closer initial attention (Sadioglu Cagdas et al., 2024). Disease activity assessment should include validated instruments such as BASDAI or ASDAS at predetermined intervals. Inflammatory markers including CRP provide complementary objective information. Physical examination documenting spinal mobility through the modified Schober test, alongside peripheral joint assessment when relevant, completes clinical evaluation. A structured monitoring timeline guides follow-up intensity. An initial assessment at 4 weeks evaluates early tolerability and identifies immediate concerns. Thorough evaluation at 12 weeks includes disease activity measurement, inflammatory marker testing, and physical examination. This time point captures the majority of clinically significant changes after switching. The final transition assessment at 24 weeks provides an important evaluation of switching success as most discontinuations and adverse events occur within this window. Patients maintaining stability through 24 weeks can resume routine monitoring schedules. Substantial increases in disease activity scores or new symptom manifestations warrant structured evaluation to distinguish true disease flares from expectation-mediated responses. True flares demonstrate concordant changes across subjective patient reports, objective examination findings, and inflammatory marker elevations, indicating genuine loss of disease control that requires therapeutic intervention (Deodhar et al., 2021). When subjective symptom increases occur without concordant changes in the inflammatory markers or physician assessments, a nocebo response should be considered. The structured approach to distinguishing this pattern from true disease breakthrough, and the appropriate clinical response to each, is addressed in the following discussion on nocebo management. When objective evidence confirms true loss of disease control, management options include optimizing the current biosimilar through dose adjustment or interval modification (Wei et al., 2020). Transitioning to an alternative biosimilar may be considered if formulation-specific issues are suspected, though evidence supporting this approach remains limited. Reverting to the reference product is appropriate when the objective efficacy loss persists without any alternative explanation. Changing to a different therapeutic mechanism, including IL-17 inhibitors, may be warranted if loss of TNF inhibitor effectiveness is confirmed through adequate biosimilar optimization attempts.

Safety monitoring focuses on detecting adverse events indicating immunogenic reactions or product-related issues. Infusion reactions for intravenous products or injection-site reactions for subcutaneous formulations should be documented (Deodhar et al., 2019). Mild local reactions occur commonly with both biosimilars and reference products and do not necessarily indicate switching problems. New occurrences or increased severity compared to the prior experience warrant further evaluation. Anti-drug antibody testing is not routinely necessary given demonstrated immunogenic equivalence between products. Testing becomes valuable when unexplained loss of efficacy occurs or when unusual adverse events indicate immunogenic mechanisms. The serum drug level and antibody testing together can inform decisions regarding dose adjustment versus product change in patients with suspected antibody-mediated drug clearance.

### 5.3 Nocebo effect recognition and management

The nocebo effect generates genuine symptom experiences through expectation-mediated neurobiological mechanisms rather than pharmacological change. In AS, chronic axial pain involves established central sensitization. Negative expectations about product transition can activate descending pain facilitatory circuits, suppress endogenous opioid analgesia, and amplify symptom perception without any change in the underlying disease activity. Long-term conditioning to a specific reference product contributes through a separate pathway. Introducing a biosimilar alters the conditioned stimulus even when the pharmacological substrate shows equivalence, thus partially extinguishing the conditioned analgesic response (Colloca et al., 2019).

Evidence-based mitigation requires moving beyond general transparency toward targeted communication strategies. Positive outcome framing, in which switching is presented as a clinically supported decision rather than an administrative measure, leads to favorable expectations and reduces nocebo-related symptom reporting. A systematic review of nocebo mitigation strategies identified positive framing as the most frequently studied approach, with over half of the relevant studies reporting significant reductions in nocebo-related outcomes when applied in verbal or written form (Car et al., 2024). Eliciting and directly addressing specific patient concerns about the manufacturing quality or immunogenic risk are more effective than delivering standardized information alone (Kristensen et al., 2018). For patients with health anxiety or catastrophizing tendencies, brief psychoeducational approaches drawing on symptom attribution retraining have shown feasibility in inflammatory arthritis populations, though formal validation within AS-specific switching programs requires further research.

When a switched patient reports symptom worsening, concurrent objective assessment is essential. Elevated inflammatory markers combined with physician-assessed clinical deterioration and patient-reported worsening indicate a true disease flare requiring therapeutic intervention. Subjective worsening accompanied by stable CRP, unchanged ASDAS-CRP, and an unaltered physician assessment indicates a probable nocebo response. In such cases, communicating objective findings directly to the patient with reassurance that inflammatory control is maintained addresses the expectation component without requiring product reversal (Rezk and Pieper, 2017). A structured follow-up within 4 weeks reinforces clinical surveillance and typically supports symptom stabilization. Product reversal or therapeutic drug monitoring should be reserved for cases where the objective disease activity change is confirmed on repeated assessment or where sub-therapeutic drug levels indicate pharmacokinetic rather than expectation-mediated worsening.

## 6 Evidence gaps and future research priorities

A foundational limitation in the current evidence base concerns the methodological constraints that are inherent to observational research. Clinicians may preferentially switch patients with more stable disease while continuing reference products for those with more tenuous control or complex treatment histories. Propensity score matching, as applied in the KOBIO registry analysis, can adjust for measured baseline differences but cannot account for unmeasured confounders such as disease severity trajectories, patient adherence patterns, or concomitant medication changes occurring around the time of switching. Furthermore, the existing registry studies do not distinguish adequately between clinician-initiated switching and administratively mandated non-medical switching. These two implementation contexts differ substantially in patient preparation and expectation management, yet their outcomes are rarely reported separately. Prospective studies with prespecified subgroup analyses comparing these implementation modes would provide more actionable guidance for healthcare systems designing switching programs.

A second gap concerns the uneven distribution of evidence across TNF inhibitor agents. The available switching data are concentrated for the infliximab biosimilar CT-P13, which is supported by the PLANETAS extension trial and multiple multinational registry analyses. Evidence for other infliximab biosimilars is derived from regulatory extrapolation based on CT-P13 comparability data rather than AS-specific switching studies. For etanercept and adalimumab biosimilars, published switching data in AS populations are sparse, with available reports drawn from mixed inflammatory arthritis cohorts containing small AS subgroups or from observation periods that are too brief to capture delayed immunogenic events. Dedicated prospective analyses focused on these agents in well-defined AS populations represent the most immediate research priority in this field.

Multiple sequential switching scenarios and long-term follow-up constitute the third area requiring investigation. Current evidence addresses single transitions from the reference product to the biosimilar under conditions of established disease stability. Whether repeated switching carries additional immunogenic or clinical risk has not been adequately examined in AS patients. Real-world trajectories involving multiple product changes are increasingly common as the biosimilar markets expand, yet their safety implications remain poorly characterized. Additionally, most registry analyses report retention and safety data extending to 4 or 5 years. Whether prolonged biosimilar exposure affects long-term disease progression or introduces late-emerging safety signals differently from continued reference product use cannot be assessed from available data. Extended follow-up from existing registry infrastructure, without requiring new trial development, would be the most efficient means of addressing this question.

Finally, evidence-based strategies for managing nocebo effects in AS switching programs remain underdeveloped. The nocebo phenomenon contributes meaningfully to switching discontinuations, as demonstrated by subjective complaint patterns observed in mandatory switching cohorts where objective disease parameters remained stable. Current clinical guidance relies on general principles of transparent communication and shared decision-making. However, no controlled studies have evaluated specific psychological or educational interventions validated in AS populations. Structured expectation management, positive outcome framing, and cognitive–behavioral approaches are theoretically supported by the broader nocebo literature but have not been tested in this clinical context. Trials examining the comparative effectiveness of these approaches would provide the evidence needed to translate general communication principles into reproducible clinical tools.

## 7 Conclusion

Biosimilar switching in AS treatment represents a scientifically supported clinical practice. It maintains therapeutic efficacy and safety in appropriately selected patients who have achieved stable disease control. Evidence from randomized controlled trials and real-world registries demonstrates that transitioning from the reference infliximab to biosimilar formulations preserves disease activity control. The transition does not introduce additional safety risks or increase immunogenic responses. These findings support theoretical predictions established through molecular and functional similarity assessments. The strength of the available evidence differs meaningfully by agent and should inform clinical confidence in individual switching decisions. The infliximab biosimilar CT-P13 is supported by the most substantial data, encompassing randomized trial and multinational registry evidence. Evidence for the other infliximab biosimilars rests on regulatory extrapolation rather than AS-specific switching studies, representing a moderate level of inferential confidence. Etanercept and adalimumab biosimilars currently lack sufficient AS-specific switching data to support broad recommendations. Clinicians should, therefore, calibrate their confidence to the evidence tier applicable to the specific agent under consideration. Special populations warrant individualized clinical assessment regardless of the agent involved. Successful implementation depends on several key principles. These include patient selection based on objective disease stability, transparent communication explaining biosimilar development and regulatory processes, and individualized monitoring protocols addressing baseline risk factors. Structured evaluation is also needed to distinguish nocebo effects from true disease activity changes when the patients report symptom worsening. Evidence-based communication strategies, including positive outcome framing and individualized concern mapping, offer practical tools for reducing nocebo-related discontinuations, though their formal validation in AS-specific switching programs remains an unmet research need. The remaining evidence gaps, including those concerning non-infliximab biosimilars, multiple switching scenarios, and nocebo effect management, do not diminish the clinical value of acting on currently available evidence. Where data are sufficient and patients are appropriately selected, biosimilar switching represents a well-supported clinical strategy. Beyond individual patient care, biosimilar adoption creates opportunities for healthcare cost savings typically ranging from 15% to 35%, thus enabling expanded treatment access for patients who are currently denied biologics due to budget constraints. Through evidence-based switching practices that combine rigorous patient selection, effective communication, appropriate monitoring, and individualized clinical judgment, clinicians can ensure high-quality treatment delivery and advance the sustainability and accessibility of biologic therapy for broader AS populations.
